# Poly (Lactic-Co-Glycolic) Acid–Poly (Vinyl Pyrrolidone) Hybrid Nanoparticles to Improve the Efficiency of Oral Delivery of β-Carotene

**DOI:** 10.3390/pharmaceutics14030637

**Published:** 2022-03-14

**Authors:** Wan-Yi Liu, Yun-Shan Hsieh, Yu-Tse Wu

**Affiliations:** School of Pharmacy, Kaohsiung Medical University, Kaohsiung 80708, Taiwan; sh980713@gmail.com (W.-Y.L.); u105003038@gap.kmu.edu.tw (Y.-S.H.)

**Keywords:** β-carotene, poly (lactic-co-glycolic) acid, hybrid nanoparticles, oral bioavailability

## Abstract

The aim of this study was to develop a nanoparticle formulation made of poly (vinyl pyrrolidone) (PVP) and poly (lactic-co-glycolic) acid (PLGA) for the oral delivery of β-carotene (BC). The hybrid nanoparticles were prepared by the interfacial deposition method, and the physicochemical properties of this formulation were characterized in terms of its morphology, particle size, size distribution, encapsulation efficiency, dissolution, intestinal permeability, and in vivo pharmacokinetics. Our results demonstrated that BC-loaded nanoformulation and PLGA nanoparticles (PNP) significantly enhanced a release 6.1 times higher than BC suspension. The fortification of PVP into PLGA nanoparticles, named PLGA–PVP hybrid nanoparticles (PPNP), significantly reduced the particle size, as well as led to an increase 1.9 times higher in the in vitro release of BC, compared with PNP. For the ex vivo intestinal permeability assessment, PNP and PPNP–K15 significantly enhanced the intestinal permeability by 2.7 and 6.5 times at the jejunum, and 2.3 and 4.5 times at the ileum, when compared with unformulated BC. According to the pharmacokinetic study, the optimized hybrid formulation significantly increased the peak plasma concentration (C_max_) and the area under the curve (AUC_0-t_), and the oral relative bioavailability showed a five-fold enhancement compared with that of the BC suspension. Our results indicate that the hybrid nanoparticulate delivery system is an efficient strategy for the oral delivery of BC.

## 1. Introduction

Carotenoids are naturally occurring pigments that can be found in various vegetables and fruits, such as carrots and spinach, and even some animal products [1]. The long-term supplementation of carotenoid-containing foods exerts beneficial effects on human health, such as lowering the incidence of cardiovascular diseases, eye disorders, and cancers, according to epidemiological studies [2]. Among these carotenoids, β-carotene (BC) is relatively abundant and has high pro-vitamin A activity. BC is a cyclic carotenoid compound containing 11 conjugated double bonds that possesses high hydrophobicity and chemical instability. The abovementioned properties limit BC’s oral absorption. In addition, the absorption of BC is affected by several factors, such as the section in the plant source, the coexistence of other food ingredients, and the style and extent of cooking processing, which result in a variable bioavailability of BC [3].

Previous studies have proposed various formulations to improve the absorption of BC. Liposomal formulations have been utilized to encapsulate carotenoids, with the results showing that the highest bioaccessibility of liposomal carotenoids occurs on lutein, followed by BC and lycopene [4]. The uptake of BC-loaded liposomes prepared with milk and soy phospholipids has been assessed using Caco-2 and coculture cell models to understand the interactions between intestinal mucus and liposomes [5]. A chlorogenic acid–lactoferrin–polydextrose conjugate has been proposed to stabilize the emulsion delivery system for BC, improving bioaccessibility [6]. Recently, lipid nanoparticles have been developed for the delivery of BC because the solid state significantly reduces the diffusion processes within the delivery system, which provides better protection and stability for BC [7].

Poly (lactic-co-glycolic) acid (PLGA) is a biocompatible and biodegradable polymer that is synthesized from lactic and glycolic acid, and has been approved by the U.S. Food and Drug Administration as an excipient for drug delivery and as a scaffold for tissue engineering [8]. Nanoparticles made of PLGA have several advantages for oral drug-delivery systems, such as better stability under gastrointestinal conditions, more efficient transport across the intestinal epithelium, and sustained release behavior [9]. Despite the outstanding properties of PLGA nanoparticles, the complete release of active ingredients from nanoparticles usually takes longer than a week [10], which might be not ideal for oral absorption via the gastrointestinal tract route. The hybrid nanoparticle delivery system composes of two or more materials with different physical/chemical properties involving organic and inorganic substance [11]. It displays unique properties through the mixture of materials owing to individual chemical structure. The hybrid system has some advantages better than non-hybrid nanoparticles, such as more rapid release and modulation of particle sizes [12,13].

In the current study, we proposed a hybrid nanoparticle delivery system consisting of PLGA and poly (vinyl pyrrolidone) (PVP) for improving the oral delivery of BC. PVP is often used as an excipient for oral drugs in the pharmaceutical industry due to its non-toxicity, biocompatibility, and complex affinity for hydrophobic drugs. As PVP with different average molecular weights possesses various physicochemical properties, the viscosity, adhesion ability, and dissolution behavior of the drug-delivery system can be modulated by using PVP of different molecular weights [14]. To examine the influence of the incorporation of PVP on BC-loaded PLGA nanoparticles, we characterized the nanoparticles with respect to their morphology, particle size, size distribution, encapsulation efficiency, dissolution, intestinal permeability, and in vivo oral bioavailability. To the best of our knowledge, the current work is the first study of BC-loaded PLGA NP hybridized with PVP as a release-rate modulator combined with the interfacial deposition method for enhanced oral drug delivery. The results acquired in this work are applicable for the development of nanoparticle-based oral-delivery systems for other carotenoid compounds that may be beneficial to human health.

## 2. Materials and Methods

### 2.1. Materials

β-carotene (purity > 97%) and poly (vinyl pyrrolidone) (PVP) K15, K30, and K90 were purchased from Tokyo Chemical Industries (Tokyo, Japan). PLGA (50:50, Mw 7000–17,000, Resomer^®^ RG 502H) was obtained from Evonik Industries AG (Essen, Germany). Poly (vinyl alcohol) (Mw 9000–10,000, 80% hydrolyzed) and sodium phosphate monobasic were obtained from Sigma-Aldrich Co. (St. Louis, MO, USA). Acetone was acquired from Echo Chemical Co. (Miaoli County, Taiwan). HPLC-grade acetonitrile was obtained from Fisher Scientific (Waltham, NJ, USA). Tetrahydrofuran (THF) and methyl alcohol were purchased from Duksan (Ansan, South Korea).

### 2.2. Preparation of β-Carotene-Loaded Nanoparticles

BC-loaded nanoformulations included PLGA nanoparticles (PNP) and PLGA–PVP hybrid nanoparticles (PPNP). PPNP were fortified with different PVP to produce three types of hybrid nanoparticles, namely PPNP–K15, PPNP–K30 and PPNP–K90. The composition of nanoformulations were shown in Table 1. The β-carotene-loaded nanoparticles were prepared via the interfacial deposition method referred to a previous study [10]. The organic phase (OP) contained PLGA (50 mg), PVP (10 mg), and BC in an organic solvent (acetone or THF), and the aqueous phase (AP) was a PVA (0.5%, *w*/*v*) solution. The OP was injected dropwise into the AP under magnetic stirring to form nanoparticles, and the organic solvent was evaporated through a rotary evaporator at 40 °C. The preparation parameters, including organic solvent, BC concentration in OP (0.2–1.0 mg/mL), and molecular weight of the added PVP (K15, K30, and K90), were examined to find an optimal formulation with the smallest size, a narrow size distribution, an adequate zeta potential, and the highest encapsulation efficiency.

### 2.3. Nanoparticles Characterization

#### 2.3.1. Particle Size, PDI, and Zata Potential

The samples were diluted 200 times with pure water to examine the particle size, polydispersity index (PDI), and zeta potential by dynamic light scattering (DLS; ELSZ-2000, Otsuka Tech Electronics Co., Osaka, Japan).

#### 2.3.2. Encapsulation Efficiency and Drug Loading

The encapsulation efficiency (EE) and drug loading (DL) was evaluated as referred to the previous study [15]. The unencapsulated BC was separated by ultracentrifuge (25,000× *g* for 30 min), and the PNP or PPNP were precipitated and located at the bottom of centrifuge tube. The supernatant was taken off and the precipitate was resuspended with DI water and diluted with mobile phase for analysis purposes [10]. The amount of encapsulated BC was determined by high-performance liquid chromatography coupled with a diode array detector (HPLC–DAD; Hitachi pump 5160, autosampler 5260 and detector 5430, Hitachi High-Tech Corporation, Tokyo, Japan). The sample separation was conducted on a Luna^®^ C18 column (250 × 4.6 mm, i.d., 5 μm; Phenomenex Inc., Torrance, CA, USA), and the mobile phase consisted of acetonitrile–methanol–THF at a ratio of 50:20:30 (*v*/*v*/*v*). The flow rate was set as 1.5 mL/min, and the injection volume was 20 μL. The BC concentration was determined at 455 nm. The EE and DL were calculated by Equations (1) and (2), respectively.

(1) $$\mathrm{EE}\left(\%\right)=\frac{\mathrm{Encapsulated}\;\mathrm{mass}\;\mathrm{of}\;\mathrm{BC}}{\mathrm{Total}\;\mathrm{mass}\;\mathrm{of}\;\mathrm{BC}\;\mathrm{added}\;\mathrm{initially}}\times 100\%$$

(2) $$\mathrm{DL}\left(\%\right)=\frac{\mathrm{Encapsulated}\;\mathrm{mass}\;\mathrm{of}\;\mathrm{BC}}{\mathrm{Total}\;\mathrm{mass}\;\mathrm{of}\;\mathrm{nanoformulation}}\times 100\%$$

#### 2.3.3. Morphological Evaluation

The morphology and appearance of the nanoparticles were examined by transmission electron microscopy (TEM; JEM-1400, JEOL Ltd., Tokyo, Japan). The sample preparation was based on a previous study [16]. Briefly, the sample was diluted 100 times with deionized water, and then, 10 μL was taken and dropped onto a carbon-plated copper mesh. After drying, the mesh was soaked in 2% (*w*/*v*) phosphotungstic acid for 5 min. Finally, the residual phosphotungstic acid was washed with deionized water, and the mesh was completely dried. The sample on the dried mesh was analyzed at 100 kV.

#### 2.3.4. Crystallinity

The crystalline property of various formulations was examined by X-ray powder diffraction (PXRD; Bruker D8 Advance, Bruker, Billerica, Massachusetts, USA). The diffraction angle was set to 5–50° with a scanning rate of 0.1°/s [17].

#### 2.3.5. Thermal Behavior

Differential scanning calorimetry (DSC; 2-HT, Mettler-Toledo, Columbus, OH, USA) was conducted to observe the thermal behavior of the BC, excipients, nanoparticles and hybrid nanoparticles [18]. The dried sample (weighing 10 mg) was put in a ceramic crucible. The samples were heated from 30 to 300 °C with a heating rate of 10 °C/min and then analyzed to detect the endothermic peak of the sample.

#### 2.3.6. Interaction between BC and the Excipient

Fourier-transform infrared spectroscopy (FTIR; IRSpirit, Shimadzu, Kyoto, Japan) was used to confirm the structure and functional group of BC in the nanoformulations. The samples were ultracentrifuged, dried, and then mixed with potassium chloride (1:100, *w*/*w*). After the process of grinding and compressing into tablets, the sample was evaluated at a wavenumber of 800–4000 cm^–1^.

### 2.4. In Vitro Drug-Release Behavior

The apparatus II paddle method (SR8-Plus, Hanson Research Corporation, Los Angeles, CA, USA) was conducted to assess the in vitro drug release behavior. The drug release was assessed under pH 1.2 and 6.8 to simulate the stomach and small intestine [10]. BC or various nanoformulations (equivalent to 2.0 mg BC) were placed into a 100 mL dissolution medium containing 1% (*w*/*v*) sodium lauryl sulfate. The temperature was set at 37 ± 0.5 °C, and the speed of the paddle was 100 rpm. A 0.5 mL sample was withdrawn at 0.25, 0.5, 1, 2, 6, 12, and 24 h. At the same time, an equal volume of dissolution medium was replenished to maintain the same volume in the dissolution chamber. The withdrawn sample was centrifuged at 21,100× *g* for 10 min, and the BC release from the formulation was determined by the HPLC–DAD method as described previously.

### 2.5. Experimental Animals

Male Sprague Dawley rats (225 ± 25 g) were purchased from BioLASCO Taiwan Co., Ltd. (Taipei, Taiwan) and used to assess the intestinal permeability and oral absorption. The experimental protocol was approved by the Institutional Animal Care and Use Committee of Kaohsiung Medical University with the approval number IACUC No. 110031. The rats were fed with sufficient feed and water, and the feeding environment was maintained at 22 ± 2 °C with a 65 ± 10% relative humidity and a 12 h light/dark cycle.

### 2.6. Ex Vivo Intestinal Permeability

The rats were sacrificed, and small-intestine sections were collected. The small intestines were washed several times with normal saline, divided into three parts (containing the duodenum, jejunum, and ileum), and then soaked in Tyrode’s solution. The ex vivo intestinal permeability experiment was conducted by the everted gut sac method with a 4 cm intestinal section [16]. The intestinal section was turned over and tied on one side. The Tyrode’s solution (300 μL) was used to fill the intestinal section, which was then tied to the other side. The filled intestinal section was immersed in 250 μg/mL of the BC suspension or various nanoformulations (5 mL) for 1 h. The concentration of BC permeated into the gut sac was determined by HPLC–DAD. The average surface area of a rat’s intestine is approximately 1.25 cm^2^ per cm [19], and the estimated average surface area of our everted gut sac model is 3.125 cm^2^. The apparent permeability coefficients (P_app_) were calculated from Equation (3) [20]:

(3) $$P_{\mathrm{app}}=\frac{\Delta Q}{\Delta t}\;\times \frac{1}{{\mathrm{AC}}_{0}}$$

ΔQ/Δt is the steady-state flux (mol/s), A is the surface area of the intestine (cm^2^), and C_0_ is the initial concentration (mol/mL).

### 2.7. Storage Stability

A total of 1.5 mL of various nanoformulations were dispersed in DI water and placed in vials. The vials were sealed and stored at 5 ± 3 °C and 25 ± 2 °C with 60 ± 5% RH, and 40 ± 2 °C with 75 ± 5% RH [21]. In order to confirm the storage stability of the nanoformulations, the BC content was quantified at 0, 7, 14, 21, and 28 days.

### 2.8. In Vivo Pharmacokinetic Study

The rats were divided into three groups, including BC suspension in 2% (*w*/*v*) carboxymethyl cellulose, PNP, and PPNP–K15, and were administered by gastric gavage at a single dose of 200 mg/kg of BC. The jugular vein catheterization method was applied to build a route for repeated blood sampling [22]. The blood samples (approximately 200 μL) were collected at 0.5, 1, 4, 6, 8, 12, 24, and 36 h, and then centrifuged at 4 °C and 4000× *g* for 10 min to acquire plasma. For the plasma extraction, an aliquot of plasma (100 μL) was mixed with trans-β-Apo-8′-carotenal (20 μL, 10 μg/mL as the internal standard) and THF (200 μL). Acetonitrile (200 μL) was then added to the mixture and vortexed for 10 min to precipitate the protein. After mixing, the plasma was centrifuged (4000× *g*, 10 min), and the supernatant was withdrawn. The extraction process was repeated twice, and the supernatant was combined, evaporated to dryness, and reconstituted (100 μL) for HPLC analysis.

The BC concentration in the plasma was determined using HPLC–DAD. The Kinetex^®^ C18 column (250 × 4.6 mm, i.d., 5 μm; Phenomenex Inc., Torrance, CA, USA) was selected as the stationary phase. The mobile phase comprised acetonitrile–methanol–THF (50:40:10, *v*/*v*/*v*), with a flow rate of 1.0 mL/min. The sample injection volume was 20 μL, and BC was monitored at 455 nm. The analysis time per sample was 20 min.

### 2.9. Statistics Analysis

The results are shown as the means ± standard deviations. Differences between the formulations were analyzed using SPSS v19 (SPSS Inc., Chicago, IL, USA). This study used a one-way analysis of variance with Tukey’s post hoc test. A *p*-value of <0.05 was considered significantly different.

## 3. Results

### 3.1. Preparation of Hybrid Nanoparticles

#### 3.1.1. Selection of the Solvent and BC Concentration

An interfacial deposition method was utilized for the production of nanoparticles, and the preparative parameters, such as the organic solvent (acetone or THF) and BC concentration in the organic phase (0.2–1.0 mg/mL) were discussed. In this study, those nanoparticles with a minimum particle size, a uniform distribution (PDI < 0.3), the highest EE, and sufficient zeta potential (value lower than −20 mV) were considered to be of optimal formulation [23,24,25].

**Figure 1:**
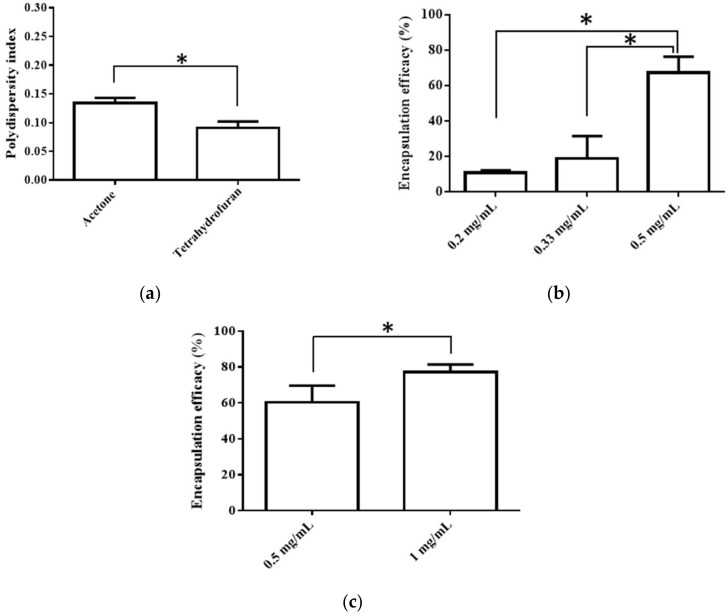
(**a**)Effects of different solvent type (acetone vs. THF) on PDI, (**b**) Effects of BC concentration (in acetone) on EE, and (**c**) Effects of BC concentration (in THF) on EE. Data are expressed as mean ± standard deviation. (*n* = 3) * indicates significant difference between two groups (*p* < 0.05).

The selection of an organic solvent for interfacial deposition is known to affect the properties of the nanoparticulate system [26]. Thus, we chose acetone, the most commonly used solvent for the interfacial deposition method, and THF, a solvent with the highest solubility for BC, for comparison. The average particle size, PDI, EE, and zeta potential were used to evaluate the quality of the resulting PNP. First, the solvent type did not affect the particle size, EE, or zeta potential when the other preparative conditions were the same, though the use of THF resulted in a more even particle distribution, as shown in Figure 1a. When acetone was used as the solvent for preparation, increasing the BC concentration (0.2 to 0.5 mg/mL) impacted the EE, and a higher BC concentration produced a better EE (Figure 1b). When THF was used as the solvent for preparation, the BC concentration of 0.5 and 1 mg/mL was evaluated owing to the higher solubility of BC (10 mg/mL) [27]. It was observed that a higher BC concentration (0.5 vs. 1 mg/mL) resulted in a better EE (Figure 1c). A comparison of the above results revealed that the optimal parameters included selecting THF as the solvent and using 1.0 mg/mL of BC to produce the nanoparticles.

#### 3.1.2. Effects of the Addition of PVP

**Figure 2:**
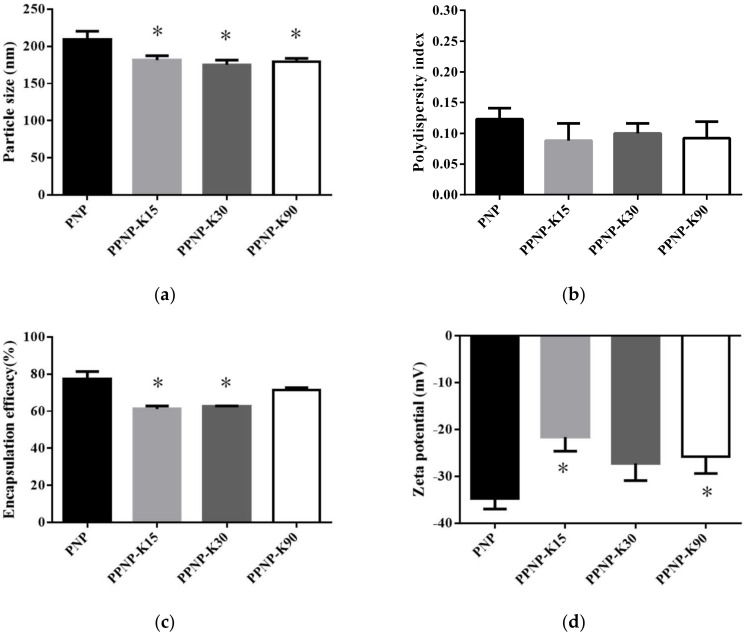
Effects of different molecular weight of PVP addition on (**a**) particle size, (**b**) PDI, (**c**) EE, and (**d**) zeta potential. Data are expressed as mean ± standard deviation. (*n* = 3) * indicates significant difference to the PNP group (*p* < 0.05).

In order to improve the biopharmaceutical properties, PVP, a hydrophilic polymer, was added to the PNP. In Figure 2 and Table 2, the results showed that the addition of PVP significantly decreased the particle size of the nanoparticles from 209 to 175 nm and did not influence the PDI. The zeta potentials of the four combinations were below −20 mV, suggesting good stability for the nanoparticles. In the aspect of EE, the addition of PVP may have a negative effect, whereas a higher molecular weight was less affected.

### 3.2. Nanoparticles Characterization

#### 3.2.1. Morphology

**Figure 3:**
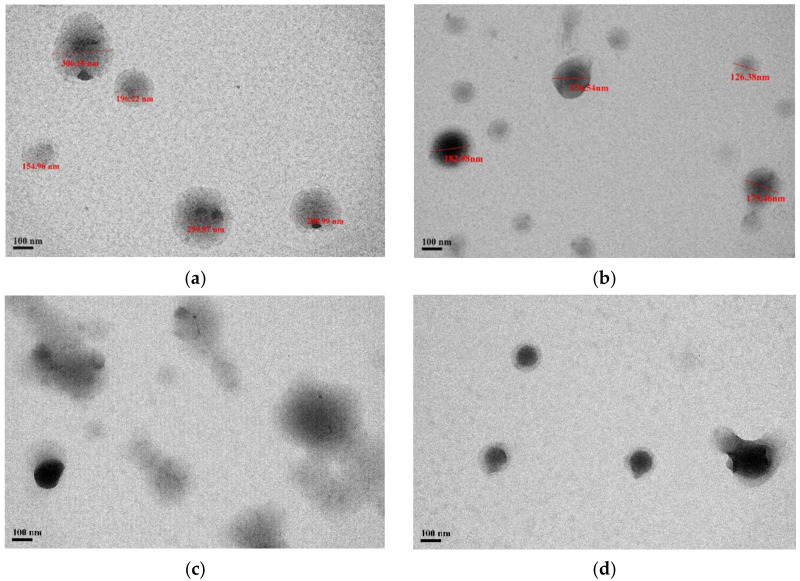
TEM images of (**a**) PNP, (**b**) PPNP–K15, (**c**) PPNP–K30, and (**d**) PPNP–K90.

As shown in Figure 3, the formulations of PNP and PPNP–K15 displayed a spherical shape with a uniform distribution, whereas the particles were partially irregular in PPNP–K30 and PPNP–K90. The particle size ranged from 155 to 306 nm and from 126 to 183 nm in the TEM images of PNP and PPNP–K15, respectively. The size-distribution results were consistent with those determined by dynamic laser scattering.

#### 3.2.2. Crystallinity

**Figure 4:**
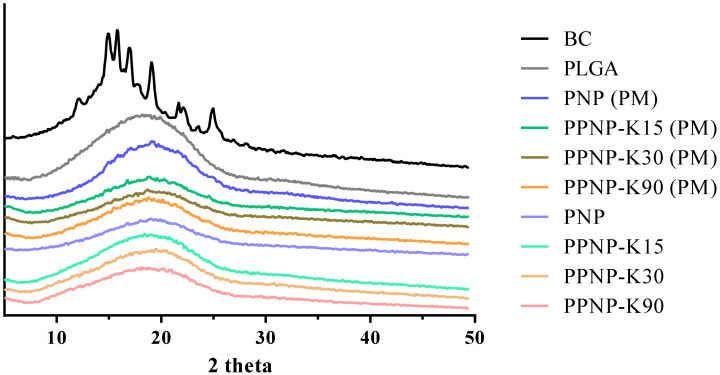
The PXRD pattern of nanoformulations. PM indicates physical mixture of nanoparticles.

The crystallinity property of these nanoformulations was examined by powder X-ray diffraction (Figure 4). The BC group had strong crystal signals at the diffraction peaks between 10 and 30°, suggesting that the BC was in a crystalline state. This phenomenon was also observed in a previous study [28]. The PLGA group had no observed diffraction peak, indicating the presence of PLGA in the amorphous state. For these nanoformulations, no peaks were observed in the PXRD pattern, suggesting that BC may exist in an amorphous state after the preparative processes.

#### 3.2.3. Thermal Behavior

**Figure 5:**
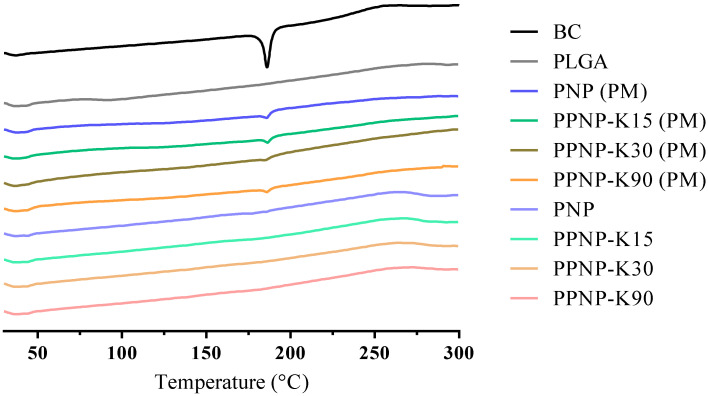
The DSC thermogram of nanoformulations. PM indicates physical mixture of nanoparticles.

The thermal performance of the nanoformulations was confirmed by differential scanning calorimetry. The endothermic peaks present the melting point of the compound or glass-transition temperature of the polymer. As shown in Figure 5, the peak of BC appeared at 186 °C, in accordance with a previous report [28]. For the physical mixture groups, endothermic peaks for BC were still observed. No obvious endothermic peaks were observed for the nanoformulations, which was in line with a previous study [29]. To sum up, the absence of melting point and no obvious signals of BC in PNP and PPNP in the spectra of PXRD (Section 3.2.2) confirm the amorphous state of BC in formulations.

#### 3.2.4. Interactions between BC and the Excipients

**Figure 6:**
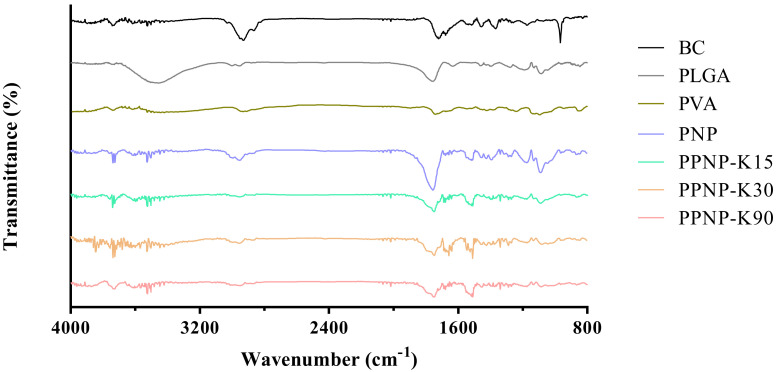
The FTIR spectra of BC, excipients and various nanoformulations.

As shown in Figure 6, the characteristic peak of BC was observed at 2949 cm^–1^ (C–H stretching), 1714 cm^–1^ (C=C stretching of conjugated alkene), 1366 cm^–1^ (β-ionone ring), and 966 cm^–1^ (C–H of conjugated alkene) [28,30]. The characteristic peak of PLGA presented at wavelengths of 2999 cm^–1^ (C–H stretching), 2955 cm^–1^ (C–H stretching), 1757 cm^−1^ (C=O stretching), and 1090 cm^–1^ (C–O stretching) [10,31]. The characteristic peak of PVA occurred at 2914 cm^–1^ (C–H stretching) and 1739 cm^–1^ (C–O stretching) [31]. For the PNP and PPNP, the characteristic peaks were similar to those of BC and PLGA, suggesting that BC should retain its major structure without being changed by the preparative processes. In addition, the FTIR spectra of the PNP and PPNP did not present the characteristic peak of PVA, as the ultracentrifugation process removed most of PVA [32].

### 3.3. In Vitro Release Behavior

**Figure 7:**
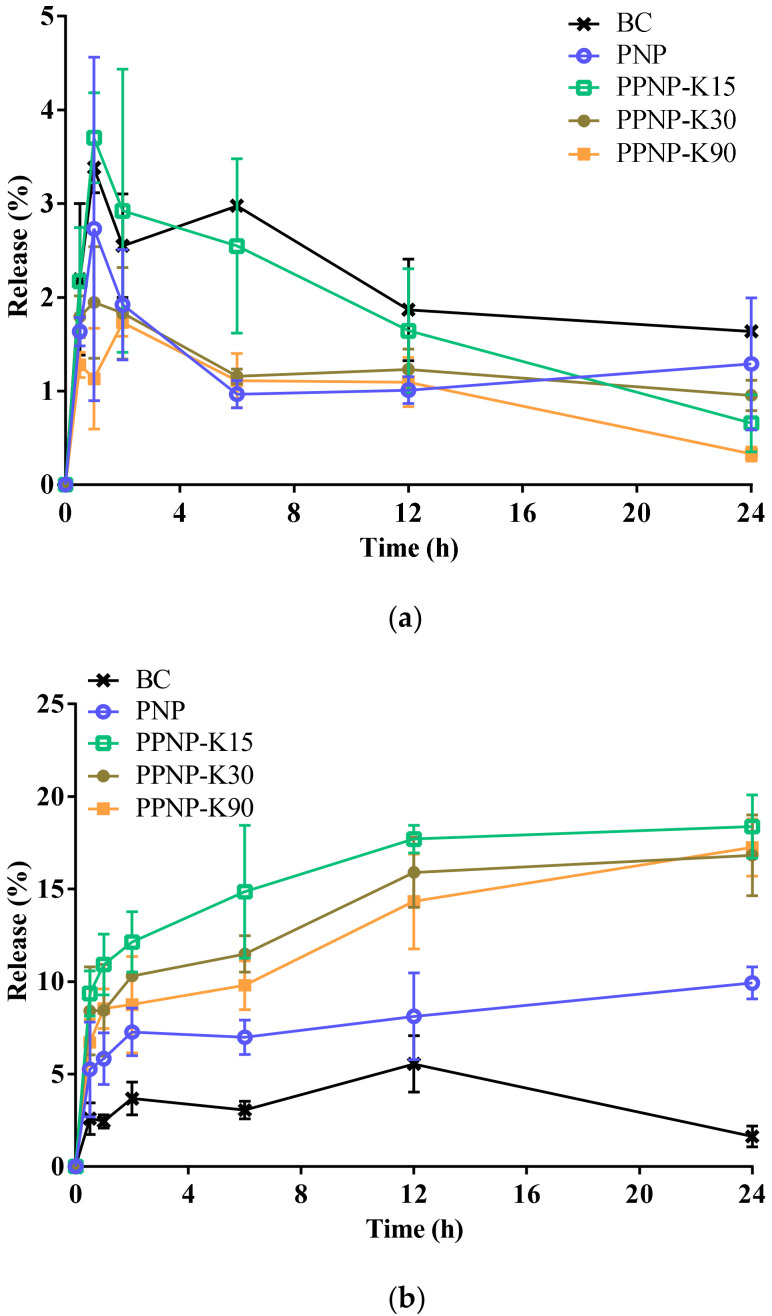
In vitro release behavior of BC, PNP, and PPNP in (**a**) pH 1.2 or (**b**) pH 6.8 condition. Data are expressed as mean ± standard deviation (*n* = 3).

The in vitro release behavior was studied in a dissolution medium with a pH of 1.2 or 6.8 to simulate the conditions of the stomach and small intestine. As shown in Figure 7a, 1.6%, 1.3%, 0.7%, 1.0%, and 0.3% BC had been released from PNP, PPNP–K15, PPNP–K30, and PPNP–K90 in pH 1.2 medium after 24 h, respectively. The release reached the peak values after 1 h (3.4, 2.7, 3.7, 2.0, 1.1% for BC, PNP, PPNP–K15, PPNP–K30, and PPNP–K90, respectively). Among the nanoformulations, PPNP–K15 achieved a highest release. In Figure 7b, it can be observed that the nanoformulations significantly enhanced the drug release, and the formulation with PVP K15 exhibited a higher and faster release profile. The strategy of applying PLGA–PVP hybrid nanoparticles can improve the solubility and dissolution behavior of BC according to our results.

### 3.4. Ex Vivo Intestinal Permeability Study

Apart from the solubility, intestinal permeability also plays an important role, affecting the oral absorption of active pharmaceutical ingredients. The concentrations of the BC suspension in the duodenum, jejunum, and ileum were 20.4 ± 2.3, 1.0 ± 0.1, and 1.0 ± 0.4 μg/mL, respectively. For PNP, the concentrations were 3.7± 0.1, 2.7 ± 0.4, and 2.2 ± 0.5 μg/mL, respectively. After the addition of PVP K15, the permeation (as indicated by concentrations) of the hybrid nanoparticles at each segment was enhanced to 2.4 ± 0.7, 6.3 ± 1.6, and 4.5 ± 0.6 μg/mL, respectively. Meanwhile, in the PPNP–K30 group, the concentrations were 3.1 ± 1.0, 2.7 ± 1.0, and 3.5 ± 1.1 μg/mL, respectively. In the PPNP–K90 group, the concentrations were 2.3 ± 1.6, 3.0 ± 1.0, and 1.9 ± 0.4 μg/mL, respectively. The estimated P_app_ values at duodenum were 7.8, 1.4, 1.0, 1.2, and 0.9 nM/cm^2^ for BC suspension, PNP, PPNP–K15, PPNP–K30, and PPNP–K90, respectively. The P_app_ values at jejunum were 0.4, 1.0, 2.4, 1.0, and 1.2 nM/cm^2^, the P_app_ values at ileum were 0.4, 0.9, 1.7, 1.4, and 0.7 nM/cm^2^ for BC suspension, PNP, PPNP–K15, PPNP–K30, and PPNP–K90, respectively. The intestinal permeation was significantly enhanced at the jejunum and the ileum with the addition of PVP K15 in nanoparticles compared with PNP.

### 3.5. Storage Stability

**Figure 8:**
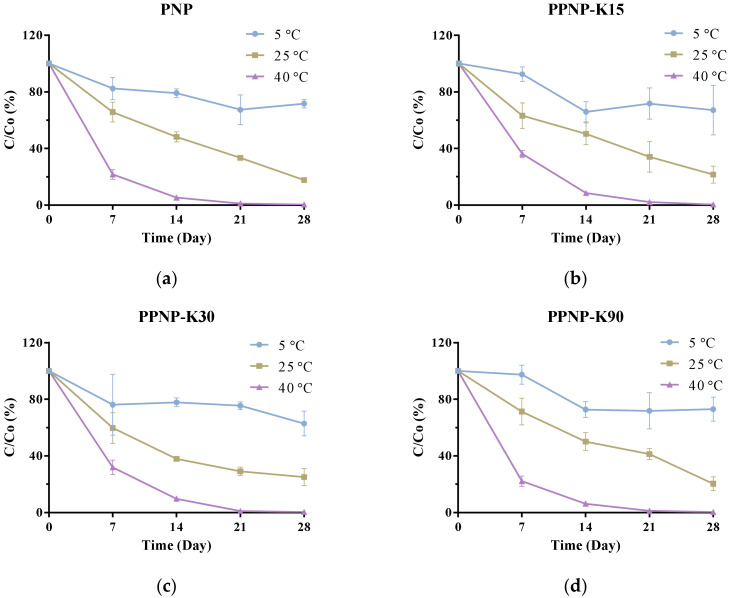
The storage stability of (**a**) PNP, (**b**) PPNP–K15, (**c**) PPNP–K30 and (**d**) PPNP–K90 stored at 5 ± 3 °C, 25 ± 2 °C, 60 ± 5% RH, and 40 ± 2 °C, 75 ± 5% RH for 30 days. Data are expressed as mean ± standard deviation (*n* = 6).

As shown in Figure 8, the drug content decreased in the examined conditions after a 30-day storage period. The BC content decreased more rapidly in the conditions of higher temperature and humidity, indicating that the encapsulation of BC into PLGA might not provide sufficient protection from temperature- and humidity-induced degradation. The BC contents remained ≥90% in the formulations containing PVP K15 and PVP K90 after seven days of storage at 5 °C.

### 3.6. In Vivo Pharmacokinetic Study

**Figure 9:**
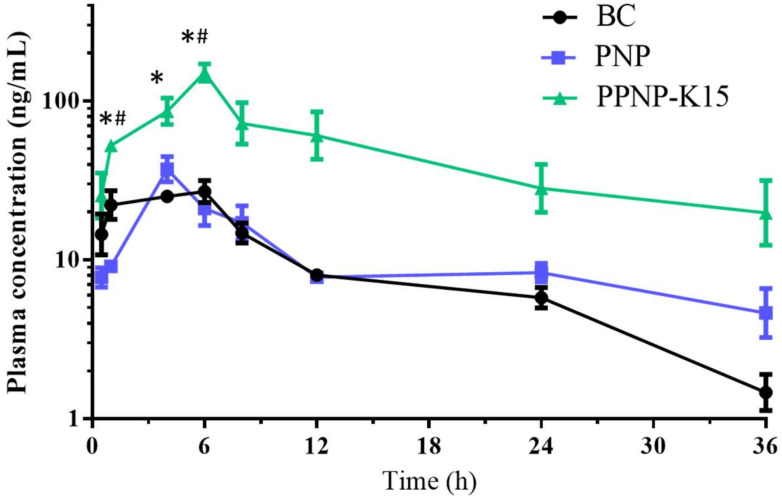
The pharmacokinetic study follows oral administration (200 mg/Kg) of BC (black), PNP nanoparticles (blue) and PPNP–K15 nanoparticles (green). Results are presented as mean ± standard error of mean (*n* = 3). * indicates significant different (*p* < 0.05) compared with the BC group. ^#^ indicates significant different (*p* < 0.05) compared with the PNP group.

An in vivo pharmacokinetic study was conducted in order to further confirm the applicability and oral bioavailability of this hybrid nanoformulation of BC. As shown in Figure 9 and Table 3, the C_max_ for PNP and PPNP–K15 was 1.39- and 5.17-fold higher than that for the BC suspension group, indicating higher absorption from the gastrointestinal tract. In addition, PPNP–K15 resulted in a significant enhancement of AUC_0–t_ from 349.9 to 1780.9 h ng/mL, and the relative oral bioavailability was improved to 508.7%. Apart from the C_max_ and AUC_0–t_, the AUC_0–12_ and AUC_0–24_ were also calculated to determine the absorption of BC. The AUC_0–12_ in the three groups was 223.3, 221.9, and 970.3 h ng/mL, whereas the AUC_0–24_ was 306.3, 318.7, and 1492.9 h ng/mL. The AUC values of the two nanoformulations were all higher than the value of the BC suspension (Figure 9).

## 4. Discussion

The aim of this study was to develop a PLGA–PVP hybrid nanoparticulate formulation to facilitate the oral absorption of BC. In our previous work, delivery by PLGA nanoparticles was shown to have a positive influence on the dissolution and intestinal permeability of fisetin, but the accumulated release reached approximately <30% after five days [10]. The strategy of blending PVP into PLGA microparticles has previously been proposed for preparing large porous particles (LPPs) to promote the release of the active pharmaceutical ingredient into the lower airways, with the results indicating that the enhancement of drug release depended on the PVP concentration [33]. LPPs are mostly applied for the fabrication of microparticulate delivery systems for the pulmonary and intramuscular routes [34,35], and we first proposed hybrid nanoparticles to facilitate the oral delivery of BC in the current study. The preparative parameters for these PLGA–PVP hybrid nanoparticles were investigated to find the optimal conditions, and the incorporation of PVP was expected to significantly boost the dissolution and oral bioavailability of BC.

In this research, the interfacial deposition method, an accessible and efficient method, was chosen to prepare the nanoformulations. The use of a rotary evaporator at 40 °C to remove organic phase within several minutes can be considered safe without obviously affecting BC’s stability. The preparation of BC-loaded solid-lipid nanoparticles also applied the rotary evaporation at 40 °C, suggesting that this method could be acceptable without significantly degrading BC [36]. Therefore, the thermal degradation of BC at 40 °C could be minimal and negligible. The use of PVA, with a molecular weight of 9–10 kDa and 80% hydrolyzation, could lower the interfacial tension more efficiently to achieve better particulate stability, and was commonly applied as the aqueous phase with a concentration of 0.5% (*w*/*v*) [37]. The selection of an organic solvent was based on a previous study where acetonitrile, acetone, and THF had been chosen as the organic phase to evaluate the properties of PLGA nanoparticles. However, acetonitrile was classified as a toxic chemical, so it was excluded. The nanoparticles prepared with THF displayed a larger size because of the relatively higher viscosity and lower diffusion coefficients in water. The results suggest that the properties of PLGA nanoparticles would be affected by the organic phase, and the particle size was consistent with that in our study [38].

Shakeri et al. [39] prepared fluorescein isothiocyanate–human serum albumin-loaded PLGA–PVP hybrid nanoparticles by nanoprecipitation. The results suggest that a higher content of PVP may result in larger particles of micrometer size. Meanwhile, this previous study did not compare the difference between nanoparticles with or without PVP. The impact of polymers with different molecular weight on the EE of active ingredients in nanoparticles has been investigated for PLGA [40,41]. The results indicated that PLGA with increased molecular weight leads to similar or relative higher EE. PVP often acts as a stabilizer because of its hydrophobic carbon chains, which extend into solvents and interact with each other to produce the steric hindrance effect for stabilizing nanoparticles [42]. In addition, PVP also acts as a dispersant due to its ability to elongate interparticle distances [43]. In the current study, the role of PVP is to facilitate the release of BC from PLGA nanoparticles, and the mechanism involved in the modulation of EE via the addition of PVP into PLGA is still unclear and may need further studies. In this study, we successfully prepared BC-loaded PLGA–PVP hybrid nanoparticles with a particle size below 250 nm and compared the differences in the properties for different molecular weights of PVP.

Although the crystal signal was not different between the nanoparticles and physical mixture groups, disappearance of the endothermic peaks of the nanoformulations was observed, suggesting that the BC had an amorphous state. The amorphous state may benefit the solubility of BC and further improve the oral absorption [38]. For the in vitro release study, dissolution methods applying non-sink conditions have become an increasingly important tool to evaluate the true performance of supersaturating formulations and drug delivery systems [44]. That is, concentration–time profiles obtained from supersaturated drug delivery systems by non-sink conditions can be recognized as a direct assessment of their ability to gain and maintain drug supersaturation [45]. The nanoformulations improved significantly the dissolution behavior under pH 6.8 conditions. The release mechanism for PPNP is dominated by PVP as it is rapidly soluble in water, generating pores on the PLGA surface and allowing water to enter the particles. Thus, relatively fast dissolution behavior for the molecularly dispersed active ingredients was observed. The release of BC declined after 1 h in the pH 1.2 medium, which might result from the acid-induced degradation of BC [46]. In addition, the release may also be restricted by the slow erosion mechanism for PLGA [47]. PLGA degrades mainly through the hydrolysis of the ester linkages between lactic acid and glycolic acid moieties and the degradation product, acidic oligomer, would build up within nanoparticles [48]. PLGA exhibits fragmentation mechanism under acidic conditions because of the crystallization of acidic oligomer, which results from low solubility at acidic pH. At acidic environments, the acidic oligomer would build up in a more homogenous way and the microenvironment within nanoparticles is close-to-uniform. On the other hand, the acidic oligomer piles up in a heterogeneous way at basic conditions, because the microenvironment varies to cause the random formation of channel on the surface and the surface erosion mechanism leads to a faster dissolution [49]. Additionally, PVP tends to form aggregates when the pH of the environment is below 2.0 due to the protonation of PVP, thus causing the destabilization of particles [50].

The duodenum is the main region for BC absorption, which is in accordance with the results found in an ex vivo intestinal permeability study [51]. A short-period examination (1 h) may give rise to even lower permeability results for nanoformulations compared with the BC suspension group. In fact, the group of hybridized nanoparticles (e.g., PPNP–K15) had significantly better permeability in the jejunum and ileum regions compared to the PNP group. The oral bioavailability was enhanced 5.1-fold for BC-loaded hybrid nanoparticles. The permeability of the PLGA nanoparticles was illustrated to be improved in some ways, such as by increased intracellular uptake via clathrin- or caveolae-mediated endocytosis, the prolongation of the GI residence time, increased absorption via M cells, and increased uptake via Peyer’s patches [9]. Thus, the PNP displayed better release and permeability compared with the BC suspension group. For the hybrid nanoparticles, PVP played an important role in oral absorption in this study. At first, the particle size significantly decreased after the addition of PVP. The particle size is a meaningful factor for the oral absorption of nanoparticles; higher translocation occurs for nanoparticles with smaller particle sizes [52]. Previous research has illustrated that nanoparticles smaller than 50 nm tend to be translocated by endocytosis, and particles sized between 20 and 500 nm are absorbed by M cells on Peyer’s patches [53]. Thus, the intestinal permeability of nanoparticles was improved in the jejunum and ileum, where M cells are mainly located. After being absorbed by Peyer’s patches, the active compound enters the lymphatic route and seems to bypass the lysosomes [53,54]. Furthermore, PVP was demonstrated to act as the porogen in the PLGA nanoparticles. The PPNP were spherical particles with large pores and improved the release behavior because of the higher surface area for erosion [33]. Moreover, the added PVP also acted as a surfactant, enhancing the solubility of the insoluble active compounds by inhibiting the precipitation in a supersaturated state, owing to the structure of the polar amide group and the nonpolar methylene and methine groups. Due to the anti-plasticizing property of PVP, the addition of PVP was indicated to increase the viscosity and reduce the diffusion of the active ingredients, preventing crystal-lattice formation. Therefore, the intestinal absorption of poorly water-soluble drugs may be enhanced [55,56,57]. Moreover, PVP in the gastrointestinal tract may be adsorbed on bile salts and reduce the critical micelle concentration of bile salt, improving the solubility after oral administration [58]. On account of the metabolism, PVP may play a role in P-glycoprotein (P-gp) and cytochrome P-450 (CYP450) modulation, whereas BC has been demonstrated to be a P-gp and CYP450 substrate [59]. Therefore, nanoparticles containing PVP may reduce the efflux and metabolism of BC, improving the oral absorption of BC [60,61].

Overall, the in vitro release behavior and intestinal permeability of BC were both enhanced after preparing PLGA–PVP K15 hybrid nanoparticles in this study. The oral absorption was also improved according to the pharmacokinetic study. Therefore, the hybrid nanoparticle system is a promising strategy for facilitating the oral delivery of BC.

## 5. Conclusions

BC-loaded PLGA–PVP hybrid nanoparticles were successfully prepared using the interfacial deposition method. The optimized nanoformulation (PPNP–K15) displayed satisfying particle properties, and the addition of PVP significantly decreased the particle size. Evaluations via DSC and FTIR indicated the formation of an amorphous state and the unchanged structure of BC after encapsulation in nanoparticles, respectively. The in vitro release and ex vivo intestinal permeability suggested better solubility and greater permeability in the jejunum and the ileum. A pharmacokinetic study further confirmed a 5-fold enhancement of the oral absorption of the PPNP–K15 group. In conclusion, PLGA–PVP hybrid nanoparticles are a practical strategy for the oral delivery of BC.

## Figures and Tables

**Table 1:** Composition of the nanoformulations.

Formulation	OP ^1^						AP ^8^ (mL)
	BC ^2^ (mg)	PLGA ^3^ (mg)	PVP–K15 ^4^ (mg)	PVP–K30 ^5^ (mg)	PVP–K90 ^6^ (mg)	THF ^7^ (mL)	
PNP	5	50	-	-	-	5	50
PPNP–K15	5	50	10	-	-	5	50
PPNP–K30	5	50	-	10	-	5	50
PPNP–K90	5	50	-	-	10	5	50

^1^ OP: organic phase. ^2^ BC: β-carotene. ^3^ PLGA: poly (lactic-co-glycolic) acid. ^4^ PVP–K15: poly (vinyl pyrrolidone)–K15. ^5^ PVP–K30: poly (vinyl pyrrolidone)–K30. ^6^ PVP–K90: poly (vinyl pyrrolidone)–K90. ^7^ THF: tetrahydrofuran. ^8^ AP: aqueous phase.

**Table 2:** Nanoparticle characterization of formulation.

Formulation	Particle Size (nm)	PDI ^1^	Zeta Potential (mV)	EE ^2^ (%)	DL ^3^ (%)
PNP	209.3 ± 11.2	0.123 ± 0.018	−34.7 ± 2.3	77.2 ± 4.2	7.16 ± 0.36
PPNP–K15	181.6 ± 5.8 *	0.088 ± 0.028	−21.6 ± 3.0	61.2 ± 1.7 *	4.85 ± 0.13 *
PPNP–K30	175.4 ± 6.1 *	0.100 ± 0.016	−27.2 ± 3.7	62.5 ± 0.4 *	4.95 ± 0.03 *
PPNP–K90	179.5 ± 4.4 *	0.092 ± 0.027	−25.8 ± 3.6	71.4 ± 1.3	5.62 ± 0.10 *

^1^ PDI: polydispersity index. ^2^ EE: encapsulation efficiency. ^3^ DL: drug loading. Data are expressed as mean ± standard deviation. (*n* = 3) * indicates significant difference to the PNP group (*p* < 0.05).

**Table 3:** Pharmacokinetic parameters following oral administration (200 mg/Kg) of BC, PNP and PPNP–K15.

	BC	PNP	PPNP–K15
C_max_ ^1^ (ng/mL)	29.2 ± 4.0	40.7 ± 4.1	151.1 ± 19.8 *^,#^
T_1/2_ ^2^ (h)	8.2 ± 0.7	27.0 ± 15.4	15.4 ± 4.5
AUC_0-t_ ^3^ (h·ng/mL)	349.9 ± 7.7	396.5 ± 42.2	1780.9 ± 525.6 *^,#^
F ^4^ (%)	-	113.3	508.7

^1^ C_max_: maximum concentration of BC in blood. ^2^ T_1/2_: half-life. ^3^ AUC_0–t_: area under curve from time 0 to 36 h. ^4^ F: relative bioavailability. Results are presented as mean ± standard error of mean (*n* = 3). * indicates significant different (*p* < 0.05) compared with BC group. ^#^ indicates significant different (*p* < 0.05) compared with PNP nanoparticles group.

## Data Availability

Data are contained within the article.
